# lncRNA NEAT1 Promotes Colorectal Cancer Progression by Increasing Inflammation

**DOI:** 10.1155/2022/4088271

**Published:** 2022-09-29

**Authors:** Qingmin Chen, Yi Qin, Min Lin, Zhao Li, Weizhong Tang

**Affiliations:** ^1^Department of Gastrointestinal Surgery, Guangxi Clinical Research Center for Colorectal Cancer, Guangxi Medical University Cancer Hospital, Nanning, Guangxi Zhuang Autonomous Region 530021, China; ^2^Department of Anesthesia, Guangxi Medical University Cancer Hospital, Nanning, Guangxi Zhuang Autonomous Region 530021, China; ^3^Experimental Research Department, Guangxi Medical University Cancer Hospital, Nanning, Guangxi Zhuang Autonomous Region 530021, China

## Abstract

**Background:**

Colorectal cancer is a digestive tract malignant tumor, ranking the second mortality and the third incidence cancer worldwide. The abnormal expression of NEAT1 is related to the occurrence and development of colorectal cancer. However, the specific mechanism of NEAT1 mediated-inflammatory pathway in the progression of colorectal cancer is still unclear.

**Methods:**

In this study, expression of NEAT1 in colorectal cancer patients was analyzed by bioinformatics. Clinical samples including peripheral blood and colorectal cancer tissues were collected for qRT-PCR, Western blot, and immunohistochemistry assay. The role of NEAT1 in the colorectal cancer progression was further confirmed by both *in-vivo* and *in-vitro* functional experiments.

**Results:**

By bioinformatics prediction, it is found that NEAT1 expression level is significantly higher in the peripheral blood of patients with colorectal cancer and is associated with poor prognosis. *In-vitro* functional studies indicated that NEAT1 knockdown suppressed the proliferation and migration of colorectal cancer cells by mediating inflammatory response. *In-vivo* tumorigenesis experiments showed that NEAT1 knockdown suppressed tumor growth.

**Conclusion:**

Abnormal high expression level of NEAT1 in colorectal cancer tissues and cells leads to poor prognosis. Mechanistically, NEAT1 triggers off the proliferation and migration of colorectal cancer cells through promoting the inflammatory reaction. Clinically, the expression level of NEAT1 in serum may be a marker for diagnosis and prognosis of colorectal cancer.

## 1. Introduction

Colorectal cancer (CRC) is one of the most common gastrointestinal cancer and the main cause of cancer-related death [1]. Recently, the development of surgery, multidisciplinary treatment model in clinical, and the implementation of new drugs, such as immunotherapy [2] and nanomedicine [3] significantly improved the therapeutic effect of colorectal cancer. However, the incidence and mortality of colorectal cancer are still high [4].

Therefore, it is in urgent need of finding new molecular markers for colorectal cancer diagnosis, treatment, and prognosis. Long noncoding RNAs (lncRNAs) are noncoding RNAs with a length of more than 200 nt [5]. Without encoding proteins function, lncRNA regulates the expression of genes at various levels [6]. Recent studies have shown that lncRNA is abnormally expressed in different cancer diseases and participating in a variety of biological functions [7], including regulating DNA methylation, histone modification, chromosome reconstruction mRNA translation as well as protein process [8–11], which plays an important role in the occurrence and development of cancer diseases [12].

NEAT1 (nuclear enriched abundant transcript 1) is an important lncRNA involved in maintaining the subcellular nuclear structure of paraspeckles [13]. It is showed that NEAT1 activates Wnt/*β*-catenin signaling and promotes colorectal cancer progression via interacting with DDX5 [14]. Zhu et al. found that NEAT1 knockdown suppresses colorectal cancer through modulating miR-193a-3p/KRAS [15]. NEAT1 also promotes the activation of inflammasomes including, NLRP3, NLRC4, and AIM2 and enhances caspase-1 activation, cytokine production, and pyroptotic cell death [16]. However, whether NEAT1 can promote the progression of colorectal cancer through inflammatory, signaling is still unknown.

In this study, we found that NEAT1 showed an abnormal high expression level in colorectal cancer tissues and cells and is associated with poor prognosis of patients. Furthermore, NEAT1 increases the proliferation and migration of colorectal cancer cells through inflammatory reaction. Therefore, NEAT1 may be a new marker and therapeutic target for colorectal cancer treatment.

## 2. Methods

### 2.1. Patient Samples

Peripheral blood and tissue samples including cancerous and normal tissues (normal tissue at least 5 cm from the tumor margin) were collected from patients who had a definite pathological diagnosis of colorectal cancer and had not undergone any treatment. Centrifuge and store at -80°C for later use. All clinical samples for this trial were obtained from the Guangxi Medical University Cancer Hospital and approved by the Ethics Committee.

### 2.2. Cell Lines and Culture Conditions

Human normal colon epithelium cell lines (NCM460) and human colorectal cancer cell lines (SW480 and HCT116) were bought from the American Type Culture Collection (ATCC, USA). Human colorectal cancer cell lines (HT29 and RKO) were purchased from the iCell (Shanghai, China). SW480 was grown in Dulbecco's modified Eagle's medium (DMEM) (GIBCO, USA). NCM460 and LOVO were grown in RPMI (1640) medium (GIBCO, USA). RKO was grown in modified Eagle's medium (MEM) (GIBCO, USA). HT29 was grown in McCOY's 5A medium (iCell, China). All cells were cultured in a constant temperature and humidified incubator at 37°C with 5% CO_2_.

### 2.3. Cell Transfection

Lenti-shNEAT1 and its corresponding negative control lentivirus were purchased from Jikai Gene (Shanghai, China) and screened for 2 weeks by addition of puromycin 8 *u*g/ml in accordance with the instructions provided for cell counting (shRNA sequence 5′–3′CACCTGTTTGCCTGCCTTCTT; NEAT1 shRNA2 sequence 5′–3′ACGCAGCAGATCAGCATCCTT).

### 2.4. RNA Extraction and Quantitative PCR

The six-hole plate was removed from the incubator, lightly rinsed with PBS, then added 1 ml TRIzol, then centrifuged with chloroform, isopropanol, and precooled 75% ethanol, respectively. The concentration and purity of RNA were determined on the instrument after the residual precipitate was mixed with 20 *u*l unenzymatic water. Then the cDNA was synthesized and quantified by reverse transcription. The Applied Biosystems 7500 qPCR (Applied Biosystems, CA) was used to carry out quantitative PCR with SYBR Green reagent (Thermo Fisher Scientific, USA).

The primer sequences designed are as follows: (see Table 1).

### 2.5. CCK-8 Assay

CCK-8 assay applied to the detection of cell viability, and CCK-8 assay kit (GLPBIO, GK10001, California, America) was used to detect cell growth at 0 h, 24 h, 48 h, and 72 h. The initial number of cells per well is 1000. The absorption value at 450 nm was tested, and then the growth curve was drawn according to the absorption value.

### 2.6. Colony Formation Assay

The cells were placed on a 6-well plate and incubated in a 37°C incubator for 2 weeks. The initial number of cells per well is 1000. After fixing with 4% paraformaldehyde for 15 min, the cells were stained with 0.1% crystal violet at room temperature for 15 min. Image J software was used to count the colonies, and the data was performed with independent sample *t* test using SPSS 25.0.

### 2.7. Wound Healing Assay

Parallel horizontal lines were drawn on the back of the six-hole plate, and the cells were evenly spread on the six-hole plate. The cut cells were removed and replaced with serum-free media. The cells were then cultured in a 5% CO_2_ incubator at 37°C for 24 h, 48 h, and 72 h, respectively, before taking pictures. The pictures were taken by ZEISS microscope. Image J software was used to count the wound closure, and the data was performed with independent sample *t* test using SPSS 25.0.

### 2.8. Apoptosis Analysis

Cell apoptosis was analyzed by the Annexin V-APC/7-AAD apoptosis kit (MULTI Science, Hangzhou, China) according to the instructions of the kit. After transfection, shRNA-HT29 cells and shNEAT1-HT29 cells were stained with Annexin V-APC and 7-AAD. The flow cytometry was performed using a Beckman CytoFLEX, and the data were analyzed using CytoFLEX Software (Beckman Coulter, California, America). Image J software was used to count the apoptosis cells, and the data was performed with independent sample *t* test using SPSS 25.0.

### 2.9. Western Blot and Antibodies

Isolated proteins were separated on a 10% sodium dodecyl sulfate-polyacrylamide gel electrophoresis (SDS-PAGE) and transferred onto a nitrocellulose membrane. The blots were incubated with antibodies (caspase-1, CST, 2225; Actin, Santa Cruz, sc-47778) overnight at 4°C. Following three washes, membranes were then incubated with secondary antibody for 1 hour in room temperature. Signals were visualized by ECL (Beyotime, China).

### 2.10. RNA Sequence

The RNA sequencing operation was carried out by Nanjing Paisenuo Biological Technology. First, RNA was extracted with TRIzol, and then the concentration and purity were detected. Further, tRNA was removed by tRNA kit, and then the RNA length was interrupted to 200-300 bp by ion interruption, followed by cDNA synthesis and subsequent analysis. After the library was built, these libraries were sequenced using Next-Generation (NGS) and Illumina-based sequencing platform, and then analyzed by software for functional annotation and path enrichment of host genes. The sequencing data has been uploaded to the NCBI database (BioProject: PRJNA872339).

### 2.11. Tumor Growth in Xenografts

Tumor growth in xenografts was carried out with the approval of the Ethics Review Committee of Guangxi Medical University Cancer Hospital. All animal research have been approved by Guangxi Medical University Cancer Hospital and were conducted in accordance with international guidelines for the maintenance and care of laboratory animals. HT29 cells transfected with shRNA were suspended in PBS and subcutaneously injected into BALB/c nude male mice (5 weeks old). The tumor volume is evaluated using a caliper based on the following formula: (*A* × *B*2)/2, where *A* is the maximum diameter and *B* is the diameter perpendicular to *A*. After 2 weeks, the nude mice were sacrificed. Tumor xenografts were then harvested from dead nude mice and weighed. Tumor xenografts were put in 4% paraformaldehyde or stored at -80°C for subsequent studies.

### 2.12. Immunohistochemistry (IHC)

After dewaxing, the slices were repaired with EDTA, then incubated with 3% hydrogen peroxide, and sealed at room temperature. Add the antibody (Ki-67, Abcam, ab16667) diluted in proportion and incubate overnight at 4°C.The secondary antibody was incubated at room temperature, then washed by PBS, DAB chromogenic solution was added to cover the tissue evenly, the color development was controlled under the microscope, and the color development was terminated by washing with pure water. The slices were counterstained with hematoxylin, taken out and washed with tap water, then differentiated with hydrochloric acid and alcohol for a few seconds, followed by three cylinders of 75% ethanol, 85% ethanol, and pure ethanol; gradient dehydration, 5 minutes for each cylinder; incubated by xylene for 5 minutes; finally sealed by neutral gum; and dried in the fume hood for microscopic examination.

### 2.13. Hematoxylin-Eosin/H&E Staining

After dewaxing, the slices were immersed in hematoxylin dye for 5 minutes, then washed with water, differentiated with hydrochloric acid and alcohol for several seconds, turned blue with tap water, and immersed in eosin dye for 5 minutes; later the slices were dehydrated with alcohol, soak in xylene to be transparent in several minutes. After cooling to dry, the slice was sealed by neutral gum and then placed in the fume hood to dry before microscopic examination.

### 2.14. Statistical Analysis

We used R version 4.1.0 to analyze the TCGA database for the corresponding results. The version of TCGA database is V29.0. All statistical analyzes were performed using SPSS 25.0 software (IBM). Data are expressed as the mean ± SD for at least three separate experiments. Unpaired two-tailed Student's *t* test between two groups was applied. Differences were considered as significant where *p* < 0.05.

## 3. Results

### 3.1. NEAT1 Expression Level Is Related to Colorectal Cancer Occurrence and Progression and Is Associated with Poor Clinical Outcome

**Figure 1:**
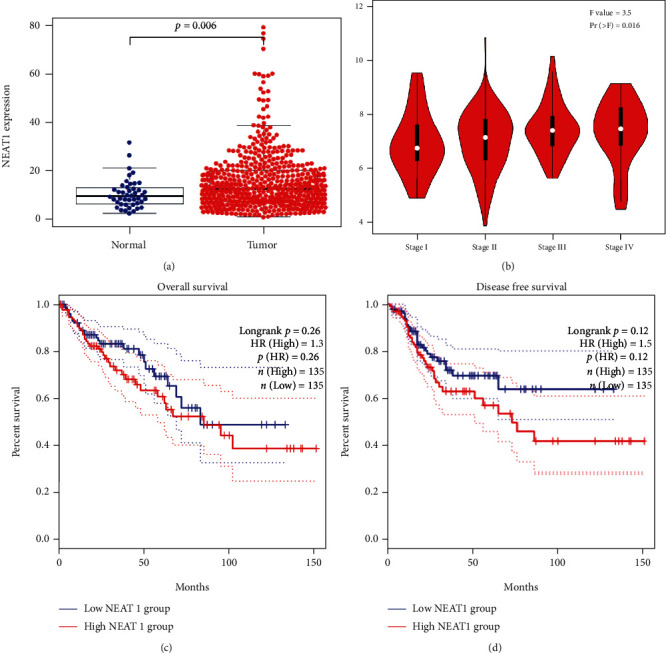
NEAT1 is highly expressed in colorectal cancer and is associated with poor prognosis of patients. (a) Analysis of expression patterns of NEAT1 in cancer and normal tissues from TCGA dataset. (b) Expression of NEAT1 in different TNM stages of colorectal cancer. (c) Kaplan-Meier analysis of overall survival (OS) of CRC patients from TCGA dataset. (d) Kaplan-Meier analysis of disease free survival (RFS) of CRC patients from TCGA dataset.

To study the correlation between NEAT1 expression and clinical significance in patients with colorectal cancer, we analyzed the expression of NEAT1 in TCGA (The Cancer Genome Atlas) database, the result of which is that the expression of NEAT1 in colorectal cancer was higher than that in normal tissues (Figure 1(a)). Furthermore, the expression of NEAT1 was positively correlated with the clinical pathological stage, namely, the higher expression of NEAT1 is in the worse pathological stage (Figure 1(b)). Survival analysis showed that the overall survival and disease-free survival of patients with high NEAT1 expression was significantly worse than that of patients with low expression of NEAT1 (Figures 1(c) and 1(d)). In summary, these results suggest that the high expression of lncRNA NEAT1 tal cancer.

**Figure 2:**
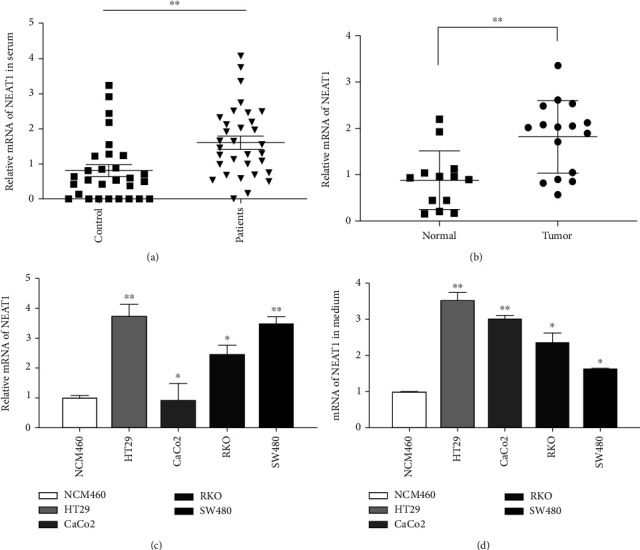
NEAT1 is highly expressed in colorectal cancer patient serum, tissues, and cells. (a) Relative expression of NEAT1 in serum of patients by qRT-PCR. (b) Relative expression of NEAT1 in CRC tissues by qRT-PCR. (c) Relative expression of NEAT1 in CRC cell lines and the normal colon epithelium cell line NCM460 by qRT-PCR. (d) Relative expression of NEAT1 in culture supernatant of CRC cell lines and the normal colon epithelium cell line NCM460 by qRT-PCR. ^∗^*p* < 0.05, ^∗∗^*p* < 0.01. The data are expressed as the mean ± S.D. of three independent experiments. Unpaired two-tailed Student's *t* test between two groups was applied.

To further verify the expression of NEAT1 in colorectal cancer, we collected clinical samples of colorectal cancer patients including peripheral blood and tissue samples as well as peripheral blood of normal people. Subsequently, we extracted RNA from peripheral blood and found that the expression of NEAT1 in peripheral blood of colorectal cancer patients was significantly higher than that of normal people (Figure 2(a)). Moreover, the expression of NEAT1 in tumor tissue was significantly higher than that in normal tissue (Figure 2(b)). Furthermore, we tested the expression of NEAT1 in colorectal cancer cell lines. The expression of NEAT1 in colorectal cancer cells HT29, RKO, and SW480 was significantly higher than that in normal cells NCM460 and so does the supernatant medium (Figures 2(c) and 2(d)). Studies have shown that tumor cells can promote the occurrence and development of tumor through the delivery of extracellular vesicles to the tumor microenvironment or peripheral blood [17]. Summarily, we found that NEAT1 expression level is related to colorectal cancer occurrence and progression and is associated with poor clinical outcome.

### 3.2. Knockdown NEAT1 Decreases Colorectal Cancer Cell Proliferation and Migration

**Figure 3:**
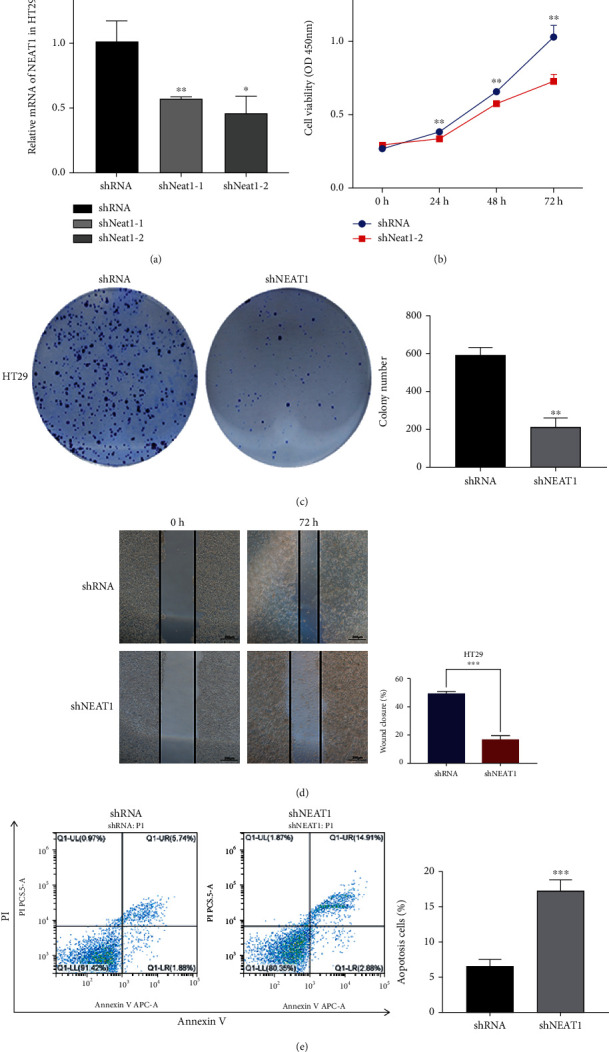
Knockdown NEAT1 suppresses the proliferation and migration of colorectal cancer cells. (a) Validation the knockdown efficacy of NEAT1 in HT29 cell line by qRT-PCR. (b) The proliferative capacity is valued by CCK-8 assay. (c) The proliferative capacity is detected by colony formation. (d) The ability of migration is represented by wound healing assay. Scale bar indicates 200 *μ*m. (e) Cell apoptosis assay by flow cytometry of HT29 cell transfected with shRNA or shRNAEAT1. ^∗^*p* < 0.05, ^∗∗^*p* < 0.01, ^∗∗∗^*p* < 0.001. The data are expressed as the mean ± S.D. of three independent experiments. Unpaired two-tailed Student's *t* test between two groups was applied.

To investigate the biological function of NEAT1 in colorectal cancer cells, a stable NEAT1 knockdown colorectal cancer cell line was established. qRT-PCR showed that the mRNA of NEAT1 was significantly lower in NEAT1 knockdown group than control (Figure 3(a)). CCK-8 assay showed that the proliferation ability decreased significantly in NEAT1 knockdown colorectal cancer cells (Figure 3(b)). The colony formation experiment showed that the number of colonies forming in the group of NEAT1 knockdown was dramatically less than that in the control group (Figure 3(c)). Wound healing assay showed that the migration ability of colorectal cancer cells decreased significantly after NEAT1 knockdown (Figure 3(d)). In cell apoptosis assay, the shNEAT1-HT29 cells exhibited significantly increased apoptotic rate when compared with cells of negative control (Figure 3(e)). In conclusion, knockdown NEAT1 decreases the proliferation and migration of colorectal cancer cells and increases the apoptosis of colorectal cancer cells.

### 3.3. RNA Sequencing Revealed That NEAT1 Regulates Colorectal Cancer through Inflammatory Response

**Figure 4:**
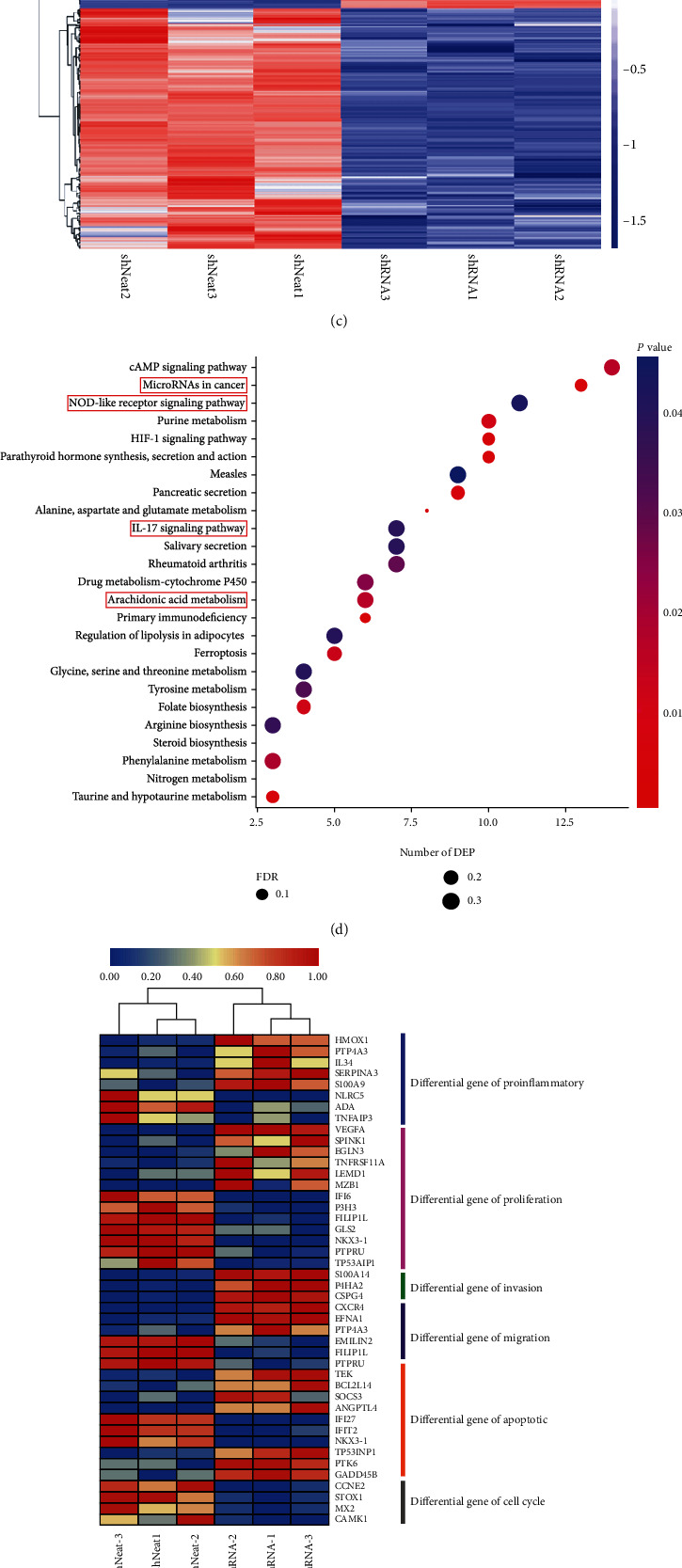
RNA sequencing reveals that NEAT1 regulates colorectal cancer through inflammatory response. (a) The expression pattern of Pearson product-moment correlation coefficient of two groups. (b) Volcanic map was used to show the different genes between the two groups. (c) Hierarchical clustering was used to show the different genes between the two groups. (d) The differential genes of all comparison groups by two-way cluster analysis. (e) The heat map of different signal pathway genes expression. (f) KEGG enrichment analysis of the differential genes. (g) Relative expression of genes in CRC cell lines by qRT-PCR. ^∗^*p* < 0.05, ^∗∗^*p* < 0.01. The data are expressed as the mean ± S.D. of three independent experiments. Unpaired two-tailed Student's *t* test between two groups was applied.

To investigate how NEAT1 involved in colorectal cancer regulation, high throughput sequencing in NEAT1 knockdown cell line was performed. The results showed that the expression pattern of Pearson product-moment correlation coefficient was similar between NEAT1 knockdown and control group, and the correlation between the two groups was high (Figure 4(a)). Then the difference between the two groups was analyzed, the result showed that NEAT1 knockdown resulted in significant changes in mRNA content. A total of 639 genes were altered between the two groups as shown in Figure 4(b), including 296 gene upregulation and 434 gene downregulation (Figure 4(b)). To explore the relationship between NEAT1 and the biological process and metabolism and signal pathway of colorectal cancer cells, we used the R package to merge the differential genes of all comparison groups and conduct two-way cluster analysis of the sample group (Figure 4(c)). The results showed that knockdown of NEAT1 could significantly change the expression level of genes involved in inflammation, cell proliferation, invasion, migration, and apoptosis (Figures 4(d) and 4(e)). KEGG enrichment analysis showed that NEAT1 participant in three inflammation-related pathways including, Arachidonic acid metabolism, IL-17 signaling, and NOD-like receptor as well as microRNAs in cancer which may involve in cell proliferation, differentiation, and apoptosis (Figure 4(f)). To further confirm sequencing data, qRT-PCR experiment was taken to test the expression level of key factors in these pathways. The results showed that expression of IL-34, VEGFA, SPINK1, S100A14, and P4HA2 was downregulated, while the expression of CCNE2 was upregulated after NEAT1 knockdown (Figure 4(g)). As a result, NEAT1 regulate inflammatory response in colorectal cancer.

### 3.4. NEAT1 Regulates Colorectal Cancer through Inflammatory Response

**Figure 5:**
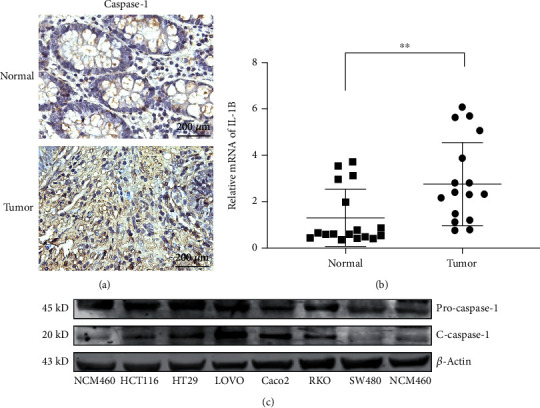
NEAT1 regulate inflammatory response in colorectal cancer. (a) Immunohistochemical (IHC) analysis of caspase-1 expression in colorectal cancer tissue. Scale bar indicates 200 *μ*m. (b) The expression of IL1B in colorectal cancer tissue by qRT-PCR. (c) The expression of caspase-1 by Western blot. ^∗∗^*p* < 0.01. The data are expressed as the mean ± S.D. of three independent experiments. Unpaired two-tailed Student's *t* test between two groups was applied.

Researchers found that NEAT1 can combine with pro-caspase-1 and promote the assembly of inflammatory bodies in respond to various inflammatory activation signals [16]. To investigate whether NEAT1 promotes the progression of colorectal cancer by promoting inflammatory response, the expression of caspase-1 in colorectal cancer tissues was detected by immunohistochemistry. The results showed that the expression of caspase-1 in tumor tissue was significantly higher than that in normal tissue (Figure 5(a)). qRT-PCR assay showed that the expression of IL1B (the downstream inflammatory factor of caspase-1) in tumor tissue was significantly higher than that in normal tissue (Figure 5(b)). Western blot showed that capase-1 expression in colorectal cancer cells was significantly higher than that in normal colonic epithelial cells (Figure 5(c)). In summary, these results further suggest that NEAT1 may promote colorectal cancer proliferation, invasion, and migration through inflammatory pathway.

### 3.5. Knockdown of NEAT1 Suppresses Tumor Growth and Inflammation-Related Gene Expression *In-Vivo*

**Figure 6:**
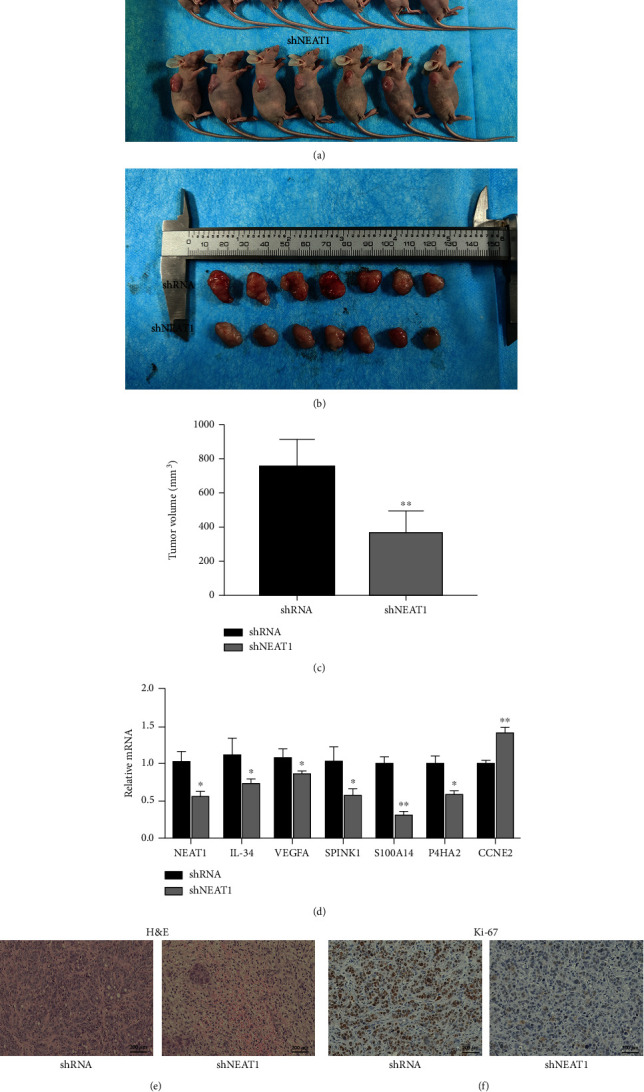
Knockdown NEAT1 attenuates tumor growth and inflammation-related gene expression *in-vivo.* (a) The tumorigenesis of nude mice. (b) The picture of tumor tissues of two groups. (c) Measurements of tumor tissue size. (d) Relative expression of genes in tumor tissues by qRT-PCR. (e) H&E staining of tumor tissues. Scale bar indicates 200 *μ*m. (f) The expression of Ki-67 in tumor tissues was analyzed by immunohistochemistry. Scale bar indicates 200 *μ*m. ^∗^*p* < 0.05, ^∗∗^*p* < 0.01. The data are expressed as the mean ± S.D. of three independent experiments. Unpaired two-tailed Student's *t* test between two groups was applied.

To further explore the role of NEAT1 in colorectal cancer progression, we selected 5-week-old male nude mice and injected cancer cells to observe their tumorigenesis. The results showed that the tumor volume of nude mice in the NEAT1 knockdown group were significantly smaller than that in control group (Figures 6(a)–6(c)). Then RNA was extracted from tumor tissue, and qRT-PCR showed that the expression of NEAT1, IL-34, VEGFA, SPINK1, S100A14, and P4HA2 was downregulated and the expression of CCNE2 was upregulated in NEAT1 knockdown tumor tissue (Figure 6(d)). H&E staining and IHC experiments showed that the malignant degree of tumor tissue decreased (Figure 6(e)), and the level of Ki-67 decreased in NEAT1 knockdown tumor tissue (Figure 6(f)). In conclusion, these results indicated that knockdown of NEAT1 inhibits tumor growth and inflammation-related gene expression *in-vivo*.

## 4. Discussion

Colorectal cancer is the third incidence and second mortality cancer around world [18]. Until now, the main basis for doctors to judge the prognosis of patients to further treatment is TNM stage, but TNM stage only considers the postoperative pathological state of patients, which often leads to great differences in the prognosis of patients with the same pathological stage [19–22]. Therefore, it is urgent to find new molecular markers for diagnosis and prognosis in colorectal cancer. lncRNA NEAT1 is a component of nuclear paraspeckle. Many studies have shown that it is associated with the occurrence and development of colorectal cancer. Luo et al. showed that long noncoding RNA NEAT1 promotes colorectal cancer progression by competitively binding miR-34a with SIRT1 and enhancing the Wnt/*β*-catenin signaling pathway [23]. Liu et al. showed that long noncoding RNA NEAT1 promotes colorectal cancer progression by regulating miR-205-5p/VEGFA axis [24]. And Yu et al. showed that lncRNA NEAT1 promotes the tumorigenesis of colorectal cancer by sponging miR-193a-3p [25]. These three studies all found that NEAT1 promoted the proliferation of colorectal cancer cells through different ceRNA signal axis. In this study, we first found a significant increase of NEAT1 expression in serum from patients with colorectal cancer. Further, we found that NEAT1 expression level is significant high in colorectal cancer and is associated with poor clinical outcome. Functional studies *in vitro* showed that NEAT1 increased the proliferation and migration of colorectal cancer cells and decreased the apoptosis of colorectal cancer cells.

Inflammation acts as a high-risk factor for cancer promotion. Chronic inflammation in human intestine can increase the risk of colorectal cancer [26–28]. Studies have shown that lncRNA CCAT1 can damage the intestinal mucosal barrier by downregulating miR-185-3p to promote the progress of Inflammatory Bowel Disease. Studies have also shown that lncRNA can promote tumor occurrence and progression by regulating the signal pathway related to inflammation. For example, lncRNA CCAT1 stimulates Inflammatory Bowel Disease Malignancy by destroying intestinal barrier via downregulating miR-185-3p [29]. To investigate whether NEAT1 promotes the progression of colorectal cancer by promoting an inflammatory response, we applied qRT-PCR, Western blot, and IHC assay, which showed that the expression of IL1B and caspase-1 increased in tumor tissues and cancer cells. RNA sequence showed that knockdown of NEAT1 inhibits inflammation-related gene expression and pathway. *In-vivo,* knockdown of NEAT1 inhibits tumor growth and inflammation-related gene expression which further confirm our proposal that NEAT1 regulated colorectal cancer progression through inflammatory response. Studies have shown that NEAT1 promotes the activation of NLRP3, NLRC4, and AIM2 inflammasomes and enhances caspase-1 activation, cytokine production, and pyroptotic cell death. They found that NEAT1 binds to pro-caspase-1 and facilitates the assembly of inflammasomes, stabilizes the mature caspase-1, and increases caspase-1 protease activity. In response to various inflammasome-activating signals, NEAT1 is released from paraspeckles and translocated to the cytoplasm to participate in inflammasome activity [16]. We hypothesize that NEAT1 may promote inflammasome in colorectal cancer to promote tumor cell growth. It is well worth for us to further explore. In summary, our study suggests that NEAT1 expression is associated with poor prognosis in patients with colorectal cancer. The expression of NEAT1 in serum may be a marker for diagnosis and prognosis of colorectal cancer. In addition to its biological importance, the study may be relevant to the clinical management of colorectal cancer patients. What is more, the results imply that targeting NEAT1-mediated inflammatory response may be a new therapeutic target for colorectal cancer treatment.

## Figures and Tables

**Table 1:** Primer sequences.

Gene name	Primer sequences(5′ to 3′)
IL34	Forward: TCCGTGTTGTCCCTCTTGAATGC
	Reverse: CACCTCACAGTCCTGCCAGTTTAG

VEGFA	Forward: AGAAGGAGGAGGGCAGAATCATCAC
	Reverse: GGGCACACAGGATGGCTTGAAG

SPINK1	Forward: TCCGTGTTGTCCCTCTTGAATGC
	Reverse: CACCTCACAGTCCTGCCAGTTTAG

S100A14	Forward: CCCATCTCATGCCGAGCAACTG
	Reverse: GCCTCTCCAGCTTCACACTCTTG

P4HA2	Forward: CCAGGAACCAAGTACCAGGCAATG
	Reverse: CTGCTCCATCCACAACACCGTATG

IFI6	Forward: GCTGGTCTGCGATCCTGAATGG
	Reverse: GAGATACTTGTGGGTGGCGTAGC

CCNE2	Forward: AATACTGACTGCTGCTGCCTTGTG
	Reverse: TACTGTCCCACTCCAAACCTGAGG

NEAT1	Forward: TTTGTGCTTTGGAACCTTGCT
	Reverse: TCAACGCCCCAAGTTATTTC

GAPDH	Forward: ACAGGGGAGGTGATAGCATT
	Reverse: GACCAAAAGCCTTCATACATCTC

IL1B	Forward: ATGAGAGCATCGAGCTTCAA
	Reverse: TGAAGGAAAAGAAGGTGCTC

*β*-Actin	Forward: GATTACTGCTCTGGCTCCTAG
	Reverse: TTGCTGTTGAAGTCGCAGGAG

## Data Availability

The raw data supporting the conclusions of this article will be available by the authors upon reasonable request.
